# Mitochondria-targeted antioxidant SkQ1 reduces age-related alterations in the ultrastructure of the lacrimal gland

**DOI:** 10.18632/oncotarget.13303

**Published:** 2016-11-11

**Authors:** Lora E. Bakeeva, Chupalav M. Eldarov, Irina M. Vangely, Nataliya G. Kolosova, Valeriya B. Vays

**Affiliations:** ^1^ Lomonosov Moscow State University, Belozersky Institute of Physico-Chemical Biology, Leninskie Gory, Moscow, Russia; ^2^ Institute of Cytology and Genetics, Siberian Branch of Russian Academy of Sciences, Pr. Acad. Lavrentieva, Novosibirsk, Russia; ^3^ Novosibirsk State University, Novosibirsk, Russia

**Keywords:** aging, dry eye syndrome, ultrastructure of lacrimal gland, mitochondria, mitochondria-targeted antioxidant, Gerotarget

## Abstract

Dry eye syndrome is an eye disorder affecting many people at an old age. Because dry eye syndrome is accelerated by aging, a useful approach to the prevention of this syndrome may be an intervention into the aging process. Previously, we showed that the mitochondria-targeted antioxidant SkQ1 delays manifestations of aging and inhibits the development of age-related diseases including dry eye syndrome. Nevertheless, the link between SkQ1's effects and its suppression of age-related changes in the lacrimal gland remains unclear. Here we demonstrated that dietary supplementation with SkQ1 (250 nmol/[kg body weight] daily) starting at age 1.5 months significantly alleviated the pathological changes in lacrimal glands of Wistar rats by age 24 months. By this age, lacrimal glands underwent dramatic deterioration of the ultrastructure that was indicative of irreversible disturbances in these glands' functioning. In contrast, in SkQ1-treated rats, the ultrastructure of the lacrimal gland was similar to that in much younger rats. Morphometric analysis of electron-microscopic specimens of lacrimal glands revealed the presence of numerous secretory granules in acinar cells and a significant increase in the number of operating intercalary ducts. Our results confirm that dietary supplementation with SkQ1 is a promising approach to healthy ageing and to prevention of aberrations in the lacrimal gland that underlie dry eye syndrome.

## INTRODUCTION

Dry eye syndrome (DES) is a frequent eye disorder affecting many people worldwide, especially at an old age [1]. DES is a multifactorial disorder of the ocular surface unit and results in eye discomfort, visual disturbances, and tear film instability with potential damage to the ocular surface and often poor quality of life. Current therapies for DES are only palliative, focusing on replacement of tear fluid to reduce the symptoms. Thus, there is a need for drugs that directly address the causes of DES. Clinical and basic studies have shown that the age-related decline of lacrimal-gland functions decreases the ability to synthesize and secrete proteins. These alterations may cause aqueous tear deficiency in DES.

Approximately 80% of the lacrimal gland is acinar cells: highly differentiated epithelial cells specialized for the synthesis, storage, and secretion of tear fluid components, such as water, proteins, glycoproteins, and electrolytes. Lacrimal glands also include epithelium-lined ducts (8%)—whose function is removal of the forming secretions into the upper conjunctival sac—as well as nerve fibers, blood vessels, and connective tissue (12%) [2]. During aging, the synthesis and secretion of proteins decrease in lacrimal glands, and acinar cells start to produce and secrete a mucous product [3-7]. The latter causes aberrations in the tear film of the eyes. Age-related alterations of the lacrimal gland have been studied in detail on a histological level. The main age-related changes in the lacrimal gland include atrophy of acinar cells, fibrosis around the ducts, expansion and enhanced tortuosity of the secretory ducts (suggestive of blockage), and an increased amount of inflammatory infiltrates containing mast cells and lymphocytes [5, 9-11]. According to histological estimates, aging of the lacrimal gland is accompanied by substantial enlargement of acini, morphological changes of acinar cells, appearance of numerous granules in acinar cells, and enlargement of the lumen in acinus ducts. Such changes in the lacrimal-gland structure during aging are believed to be the result of Harderian-gland-type metaplasia (Harderianization) of the lacrimal gland [12-16].

Ultrastructural studies on changes in the lacrimal gland during aging are scarce. Draper and coauthors [3, 4] described morphological changes in the rough endoplasmic reticulum (ER) and Golgi apparatus of acinar cells in aging lacrimal glands of Sprague-Dawley rats at different ages. Kawashima and coworkers [17, 18] compared ultrastructure of mitochondria in acinar cells of 6- and 12-month-old *ad libitum* fed Fischer 344 rats and uncovered occasional mitochondrial swelling, disorientation, shortening, and disorganization of cristae in the 12-month-old animals.

Mitochondria, when dysregulated, are a major source and target of oxidative stress. Mitochondrial dysfunction strongly promotes aging and the pathogenesis of age-related diseases including eye diseases. Some authors demonstrated a connection of age-related alterations in the lacrimal gland with oxidative stress. Other authors showed the possibility of interventions (e.g., calorie restriction) aimed at reducing excessive production of reactive oxygen species (ROS) to prevent disturbances in the mitochondrial ultrastructure of acinar cells in the lacrimal gland [17, 18]. Changes in signaling pathways associated with age-related upregulation of oxidative stress have been detected in the aging lacrimal gland [19, 20]. Increased oxidative stress can result from reductions in insulin secretion and parasympathetic signaling accompanied by an increase in hormone resistance and by accumulation of advanced glycation end products in the aging lacrimal gland [6, 19, 21].

Thus, an increasing body of evidence suggests that prevention of upregulation of mitochondrial ROS is important for possible therapeutic strategies to delay age-associated alterations and to prevent age-related disorders in humans. Despite the disappointing effects of antioxidants in clinical trials, there is growing evidence of beneficial effects of mitochondria-targeted antioxidants during aging and in age-related diseases. Previously, we showed that mitochondria-targeted antioxidant 10-(6′-plastoquinonyl) decyltriphenyl phosphonium cation (SkQ1) [22] ameliorates the signs of aging and inhibits the development of such age-related diseases as cataract, age-related macular degeneration, and glaucoma in rats [23, 24]. SkQ1 (under the brand name Visomitin) in the form of eye drops is already manufactured and has been successfully used since 2012 for treatment of DES in Russia [25, 26]. Nevertheless, the link between SkQ1's effects and its suppression of age-related aberrations in the lacrimal gland has not been explored. The aim of this study was to examine the effects of long-term dietary supplementation with SkQ1 on age-related deterioration of lacrimal-gland ultrastructure in Wistar rats.

## RESULTS

### Assessment of ultrastructural age-related alterations in the lacrimal gland

**Figure 1:**
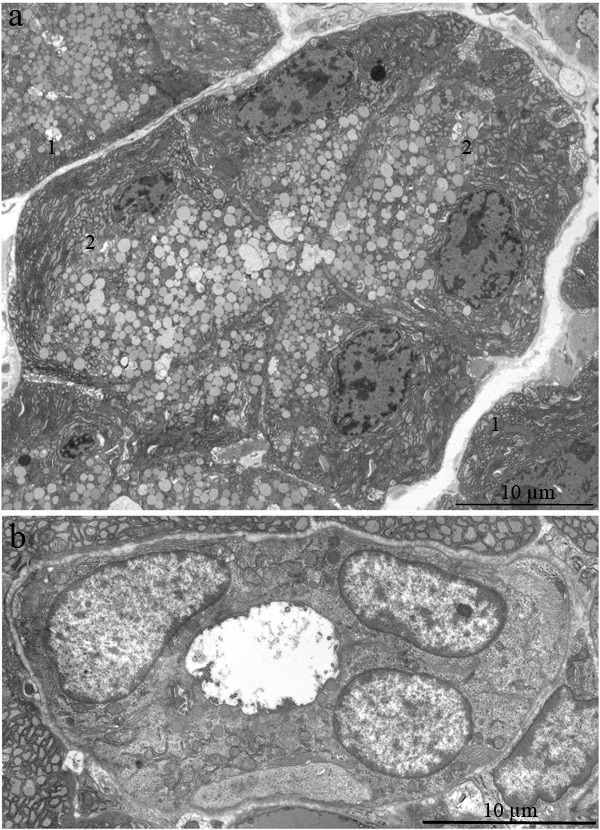
Ultrastructure of the lacrimal gland of Wistar rats at age 3 months **A.** The rosette of acinar cells: acini. Arrows “1” indicate the ER; arrows “2” indicate secretory granules in acinar cells; **B.** The ultrastructure of an intercalary duct.

**Figure 2:**
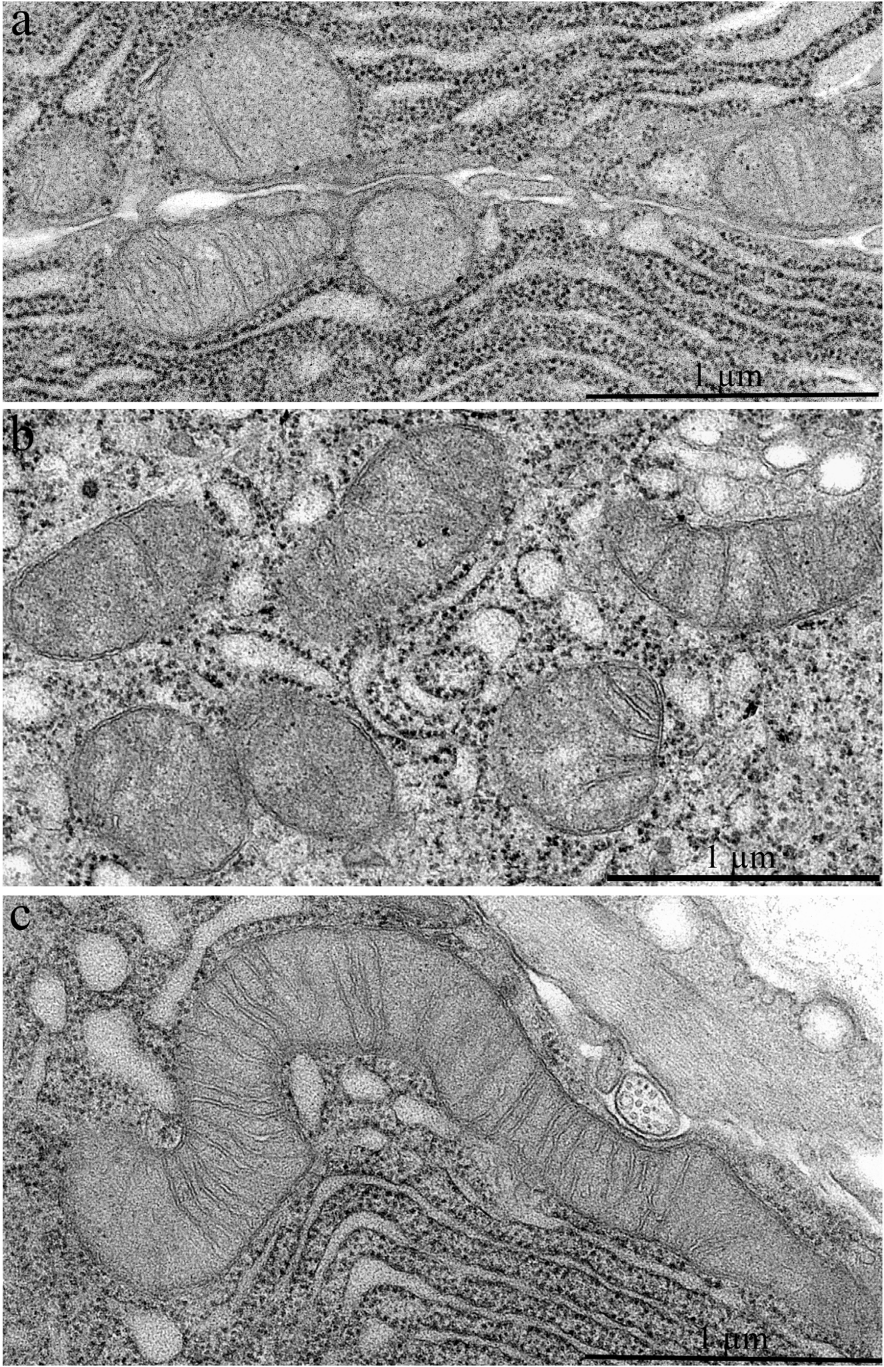
Mitochondrial ultrastructure in acinar cells in the lacrimal gland of Wistar rats **A.** 3-Month-old, **B.** 24-month-old untreated rats, and **C.** 24-month-old rats that received 250 nM SkQ1.

**Figure 3:**
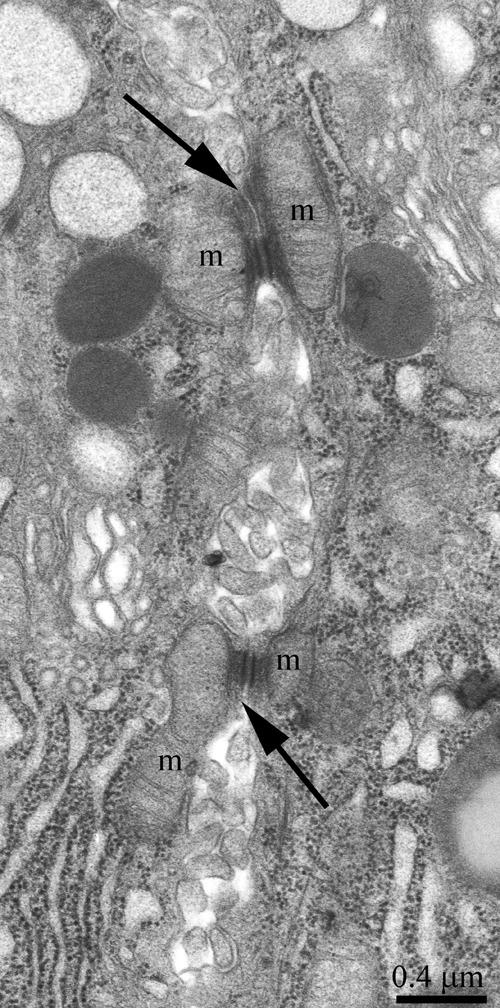
Ultrastructure of an intercellular junction complex formed by desmosomes and mitochondria that were tightly adjacent to tonofilaments on the side of each contacting acinar cell The arrows show desmosomes; m: a mitochondrion.

The ultrastructure of the lacrimal gland varied significantly with age. At 3 months of age, ultrastructure of acini and intercalary ducts conformed to the classical ultrastructural concepts of the lacrimal gland (Figure 1a, 1b). Figure 1a depicts the acinar cells, which were arranged in groups of 6-8 and formed acini that looked like a rosette with a clear center on a cross-section. Nuclei of the acinar cells were round and located near the basal membrane. The basal region of the cells was filled with the ER closely surrounding the nucleus (arrows “1” in Figure 1a). In the tight layers of the cytoplasm between the ER membranes, there were mitochondria with a small number of cristae and a light-gray matrix (Figure 2a). The apical region of the acinar cells was filled with many secretory vesicles with contents of varying electron density (arrows “2” in Figure 1a). In the literature, these structures are defined as secretory granules. We found that neighboring acinar cells in acini formed special connecting intercellular junction complexes involving desmosomes and mitochondria that were tightly adjacent to tonofilaments on the side of each contacting cell (Figure 3). In the duct cells, the cytoplasmic contents looked inconspicuous against the background of unusually high-contrast organization of the acinar cells (Figure 1b).

**Figure 4:**
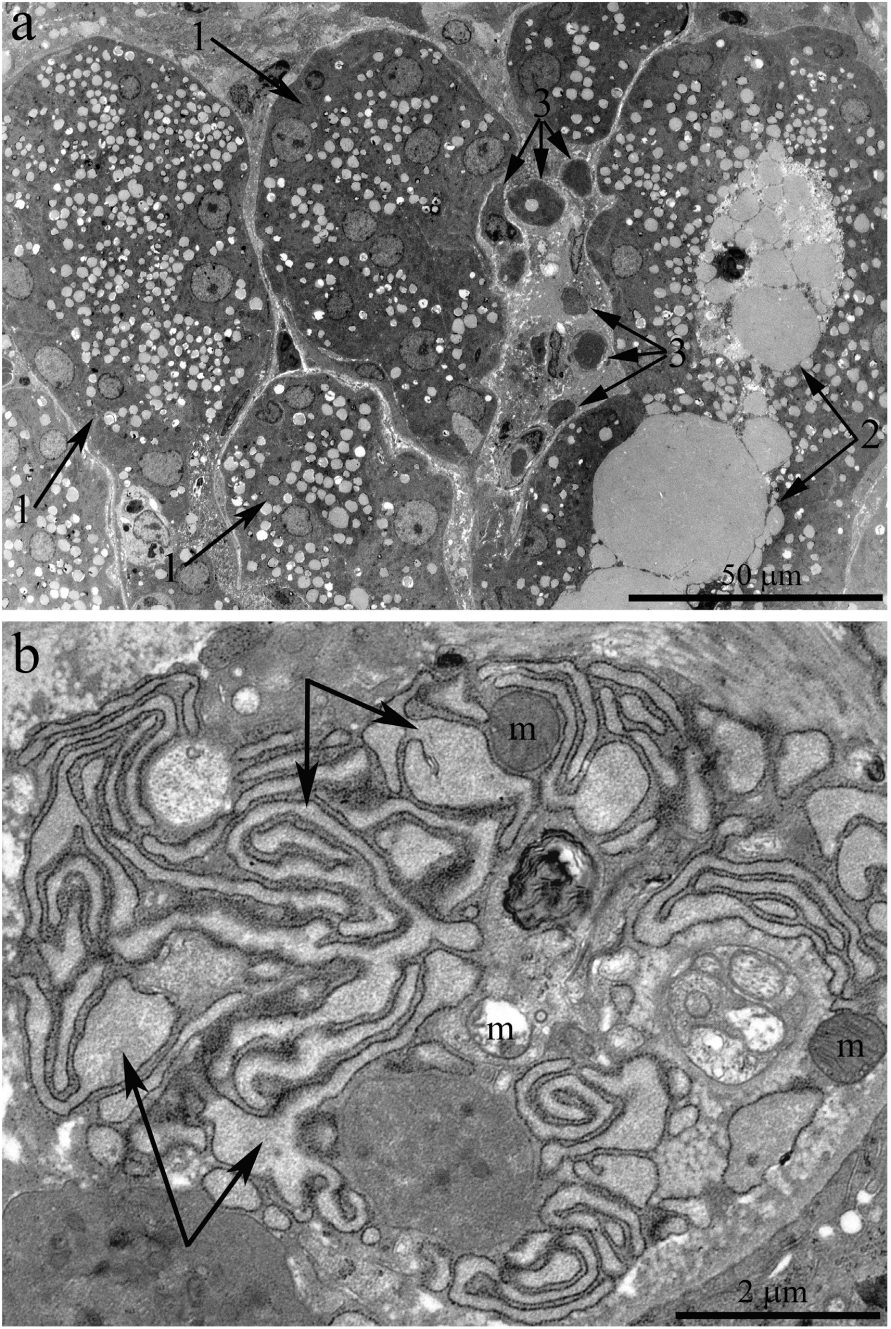
Ultrastructure of the lacrimal gland of Wistar rats at age 24 months **A.** The general appearance. One can see that the lacrimal gland is filled with enlarged ducts, whose epithelial cells show only nuclei and numerous membrane-bounded granules (arrows “1”). Arrow 2 indicates the lumen of a duct containing numerous electron-dense granules. Arrow “3”: degraded acinar cells. **B.** Ultrastructure of a degrading acinar cell in the lacrimal gland of a Wistar rat at age 24 months (at high magnification). The arrows indicate swollen cisternae of the ER; one can see partially destroyed swollen mitochondria (m).

**Figure 5:**
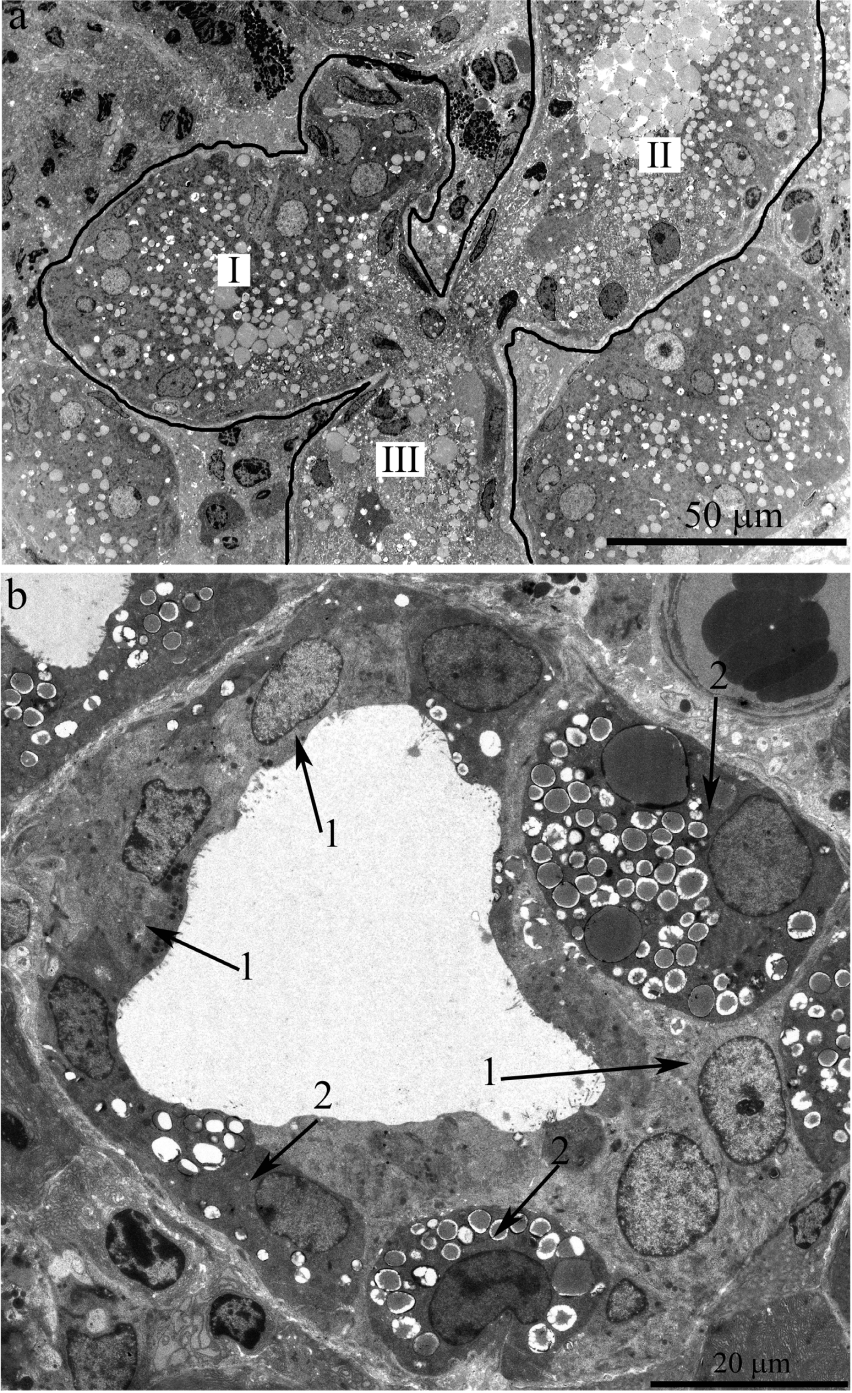
Ultrastructure of the lacrimal gland of Wistar rats at age 24 months **A.** The general appearance of a duct with complex branching: there are three branches from the main trunk of the duct in the form of paddles (labeled with Roman numbers) sharing a common center. **B.** A duct with only partially altered ultrastructure of the lining epithelial cells. Arrows “1”: epithelial cells whose ultrastructure is characteristic of epithelial cells of lacrimal-gland ducts in young Wistar rats. Arrows “2”: electron-dense cells with difficult-to-distinguish cytoplasmic contents and numerous membrane-bounded big granules.

In 24-month-old rats, a large proportion of the area of lacrimal-gland sections was filled with sections of the ducts (Figure 4a). We observed not only an increased size of the ducts but also clearly defined branching of the ducts (Figure 5a). For instance, in Figure 5a, the whole visual field of the photograph is occupied by a segment of a duct with a complicated branching pattern. One can see three branches (in the form of a trefoil, labeled with Roman numbers). These branches had one common center (schematically shown in Figure 3a).

The ducts also underwent significant ultrastructural changes. Figure 4a shows the typical ultrastructural picture of lacrimal-gland ducts in 24-month-old Wistar rats. Only nuclei and numerous membrane-bounded granules were clearly seen (Figure 4a, arrows “1”). The lumen of some ducts was filled with electron-dense granules, sometimes of considerable size (indicated with arrows “2” in Figure 4a). Meanwhile, we also observed ducts with only partially altered ultrastructure of the lining epithelial cells. Figure 5b shows a duct containing epithelial cells with the ultrastructure characteristic of the epithelial cells of lacrimal-gland ducts in young Wistar rats (Figure 5b, arrows “1”). Namely, the nuclei are readily distinguishable against the background of the light-colored cytoplasmic contents. But, as shown in Figure 4, in certain epithelial cells of this duct, the ultrastructural alterations already took place and led to transformation into electron-dense cells with barely distinguishable cytoplasmic contents and numerous membrane-bounded large granules. We believe that the ultrastructural state of this duct reflects age-related aberrations of duct ultrastructure. Apparently, the ultrastructural alterations gradually extended to all epithelial cells lining the duct; this process eventually led to the above-mentioned pattern of duct ultrastructure in the lacrimal glands of 24-month-old Wistar rats, when significantly enlarged branched ducts fill the space previously occupied by acini. The remnants of degraded acinar cells ended up squeezed on all sides by the enlarged ducts (Figure 4a, arrows “3”). Figure 4b depicts a region of a degrading acinar cell at high magnification. One can see swollen cisternae of the ER and partially destroyed swollen mitochondria. Meanwhile, the lacrimal gland of 24-month-old rats contained small numbers of acini with native ultrastructure. The ultrastructure of mitochondria in these acinar cells slightly changed relative to the mitochondria of acinar cells at 3 months of age (Figure 2b).

### Effects of SkQ1 on the age-related ultrastructural alterations in the lacrimal gland

**Figure 6:**
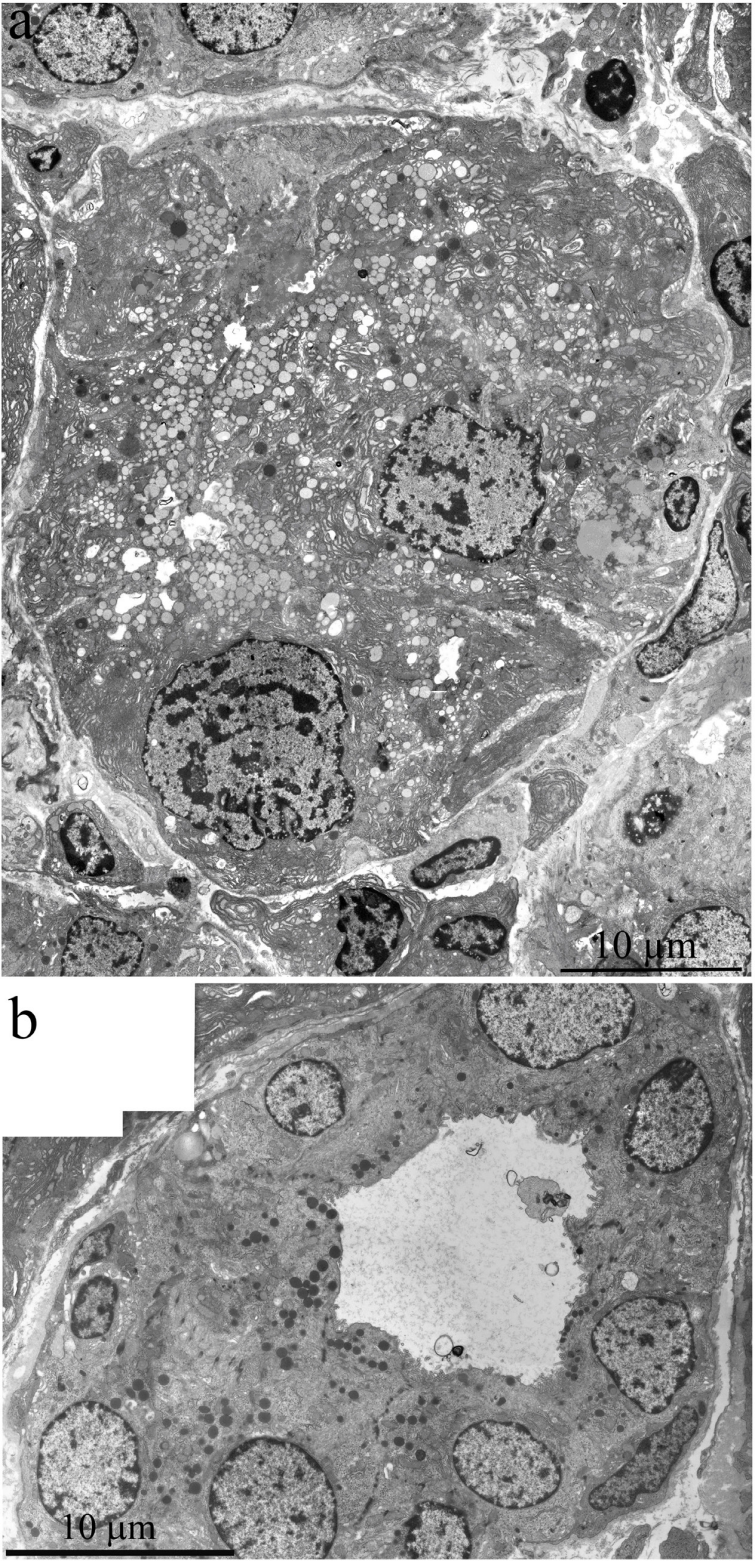
Ultrastructure of the lacrimal gland of a 24-month-old Wistar rat that received the antioxidant SkQ1 starting at age 1.5 months at the dose 250 nmol/(kg body weight) daily **A.** A rosette of acinar cells; **B.** ultrastructure of an intercalary duct. In the cytoplasm of the epithelial cells, one can see many electron-dense granules.

Ultrastructure of the lacrimal gland in 24-month-old Wistar rats treated with SkQ1 since age 1.5 months was significantly different from that in untreated Wistar rats of the same age. In the latter rats (no SkQ1 treatment), it is difficult to identify a single acinar cell in the tissue slices, but in the SkQ1-treated animals, acini were present everywhere in the slices. Figure 6a shows ultrastructure of lacrimal glands that was typical of 24-month-old Wistar rats that received the antioxidant SkQ1. Nuclei of acinar cells were located near the basal membrane of the cells, and most nuclei retained the round shape. The cytoplasm of the acinar cells was completely filled with ER membranes and numerous secretory granules with the contents of varying electron density, as in the acinar cells of the young rats. The special ultrastructure of intercellular junctions (which we observed in the young rats) was preserved.

Figure 6b shows a duct of an SkQ1-treated 24-month-old Wistar rat: the ultrastructure of epithelial cells is consistent with that in intercalary ducts in 3-month-old Wistar rats. In contrast to the young animals, there were many electron-dense granules in the cytoplasm of the epithelial cells at age 24 months. SkQ1 had a marked effect on mitochondrial ultrastructure in acinar cells of the lacrimal gland in 24-month-old Wistar rats treated with SkQ1 (Figure 2c). SkQ1 caused lengthening of mitochondria, with a simultaneous small increase in crista number.

**Figure 7:**
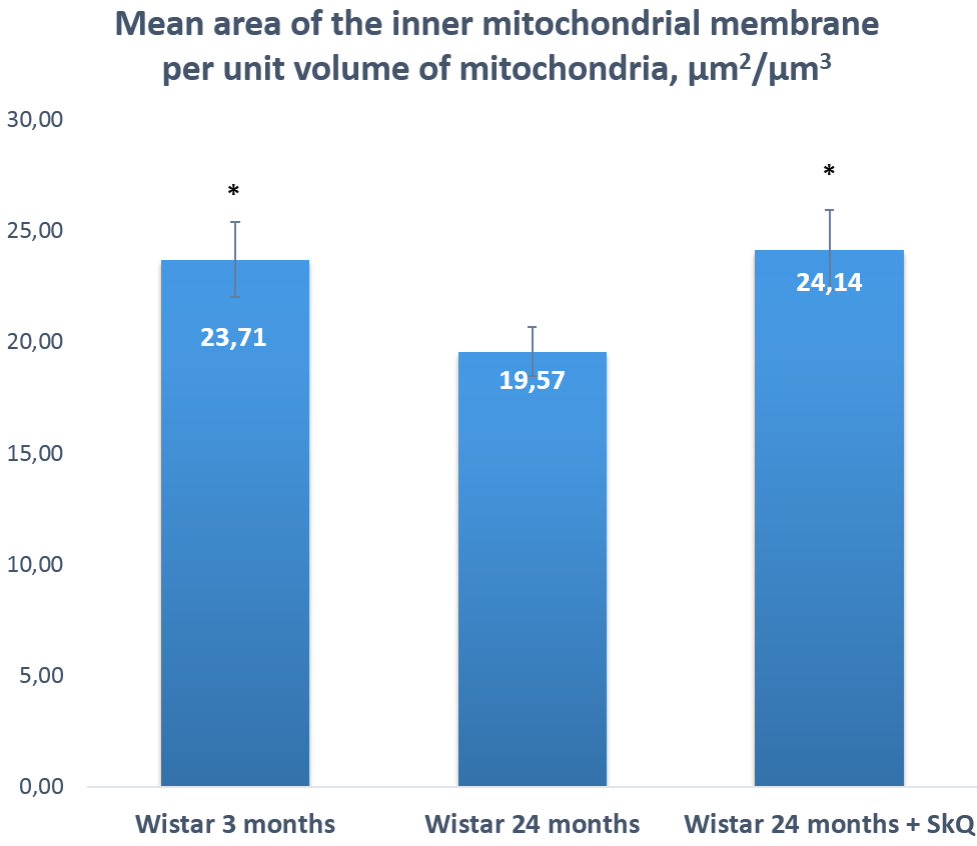
The area of the inner mitochondrial membrane per unit of volume of mitochondria in the lacrimal-gland tissue of Wistar rats at ages 3, 24, and 24 months; the latter group received the antioxidant SkQ1 (starting at age 1.5 months at the dose 250 nmol/ [kg body weight] daily) The data are presented as mean ± SEM; *p < 0.05 compared to 24-month-old untreated Wistar rats.

**Figure 8:**
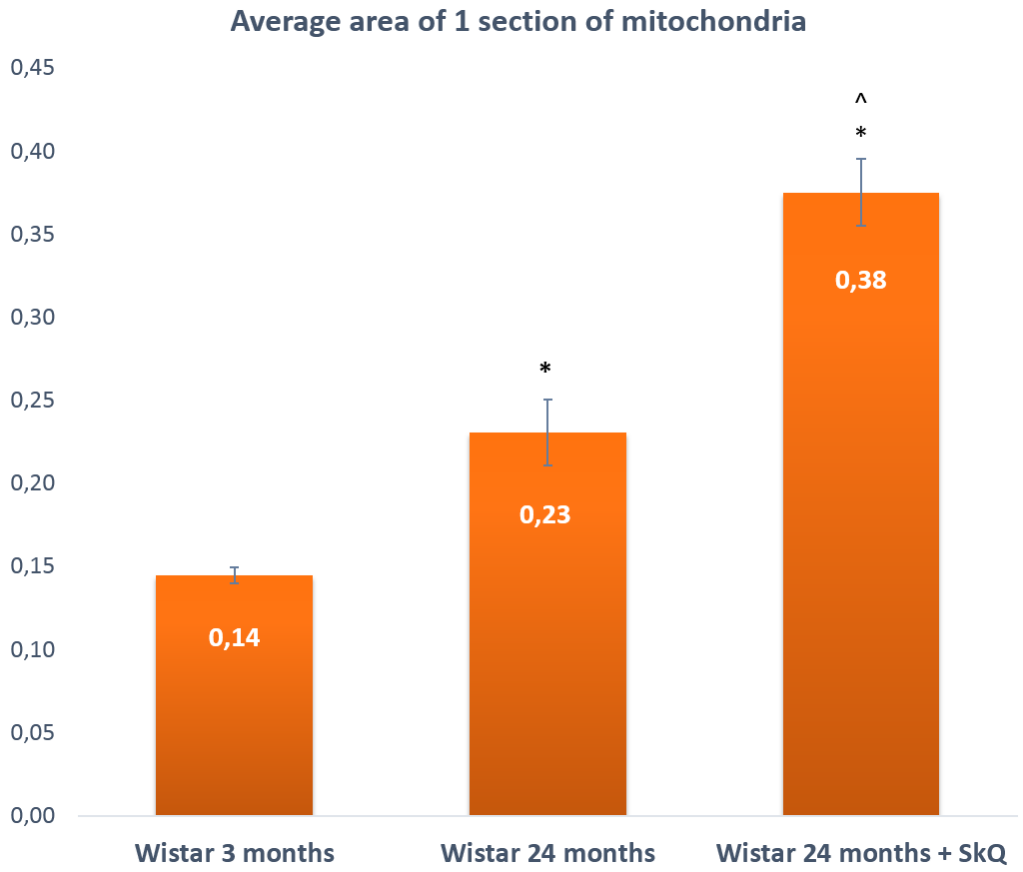
The area of one section of a mitochondrion in the lacrimal-gland tissue of Wistar rats at ages 3, 24, and 24 months; the latter group received the antioxidant SkQ1 (starting at age 1.5 months at the dose 250 nmol/ [kg body weight] daily) The data are presented as mean ± SEM; *p < 0.05 compared to 3-month-old untreated Wistar rats, ^*p* < 0.05 compared to 24-month-old untreated Wistar rats.

Two morphometric parameters of lacrimal-gland mitochondria were measured in addition to electron microscopic analysis: the average area of mitochondria in each group of animals and the ratio of the “inner membrane + cristae” area to the mitochondrial unit volume (Figures 7 and 8). The ratio was 23.71 ± 1.19 μm^2^/μm^3^ (mean ± SEM) in 3-month-old Wistar rats and 19.57 ± 1.66 μm^2^/μm^3^ in 24-month-old Wistar rats. Treatment with the antioxidant SkQ1 for 5 months prevented this decrease, resulting in the ratio 24.14 ± 1.58 μm^2^/μm^3^ at 24 months of age. The differences were significant (*p* < 0.05).

The average area of mitochondria, on the contrary, appeared to increase with age: from 0.14 ± 0.01 μm^2^ at 3 months of age to 0.23 ± 0.03 μm^2^ in 24-month-old rats, but this difference was not significant. In rats treated with SkQ1, we observed more than a twofold increase (to 0.38 ± 0.03) at age 24 months. The difference was significant (*p* < 0.05).

**Figure 9:**
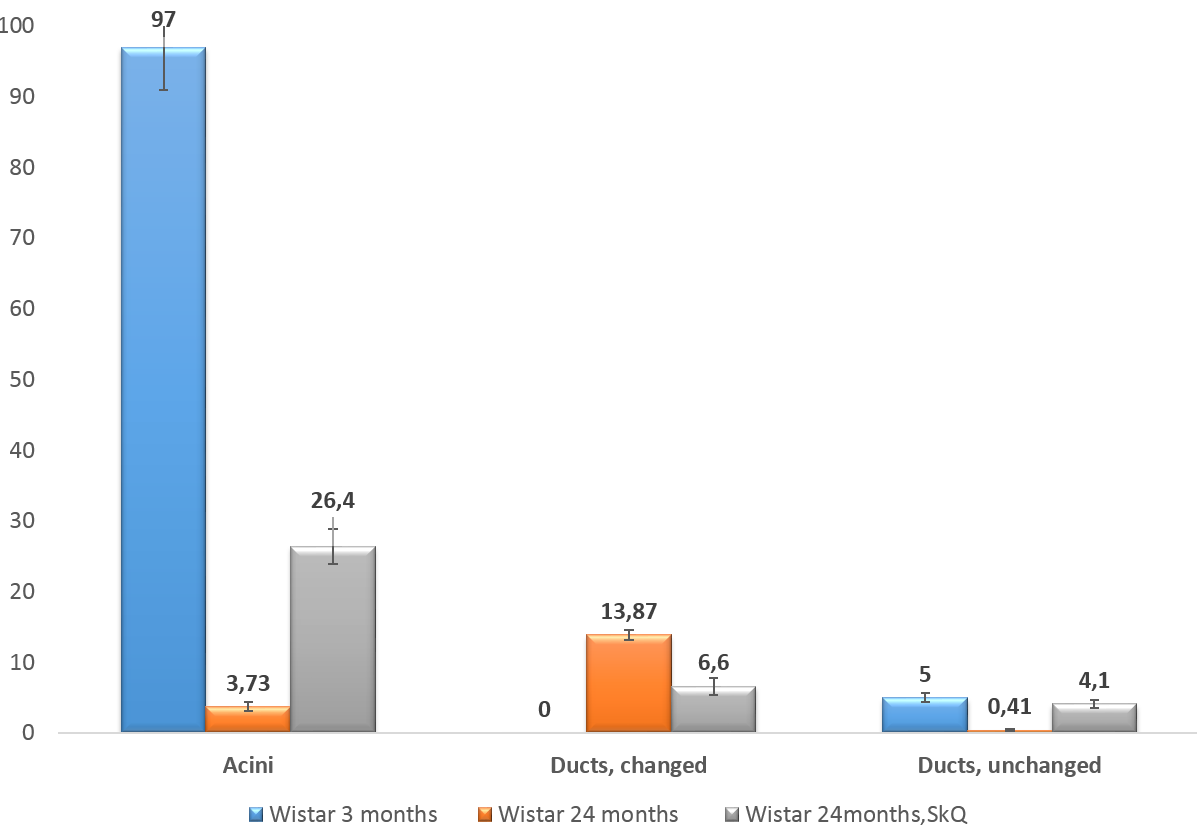
The numbers of sections of acini and of altered and unaltered intercalary ducts per unit of area (0.23 mm) in the lacrimal-gland tissue of Wistar rats at ages 3, 24, and 24 months; the latter group received the antioxidant SkQ1 (starting at age 1.5 months at the dose 250 nmol/ [kg body weight] daily) The data are presented as mean ± SEM.

The effectiveness of SkQ1 was also confirmed by morphometric analysis of electron-microscopic images of lacrimal-gland tissue (Figure 9). The numbers of acini and intercalary ducts with or without ultrastructural changes were calculated. In the lacrimal-gland tissue of 3-month-old Wistar rats, the number of acinus sections per area of 0.23 mm^2^ was 97 ± 6.09, and that in intercalary ducts was 5 ± 0.6 (Figure 9). The same parameters were assessed in 24-month-old Wistar rats: per area of 0.23 mm^2^, the number of acinus sections was 3.73 ± 0.63, the number of intercalary ducts with signs of degradation 13.87 ± 0.75, and the number of unchanged intercalary ducts was 0.41 ± 0.15 (Figure 7). In 24-month-old Wistar rats that received SkQ1 at the dose 250 nmol/(kg body weight) daily with food (starting at age 1.5 months), these data were as follows. Per area of 0.23 mm^2^, the number of acinus sections was 26.4 ± 2.44, the number of intercalary ducts with signs of degradation was 6.6 ± 1.18, and the number of unchanged intercalary ducts 4.1 ± 0.51 (Figure 9).

## DISCUSSION

**Figure 10:**
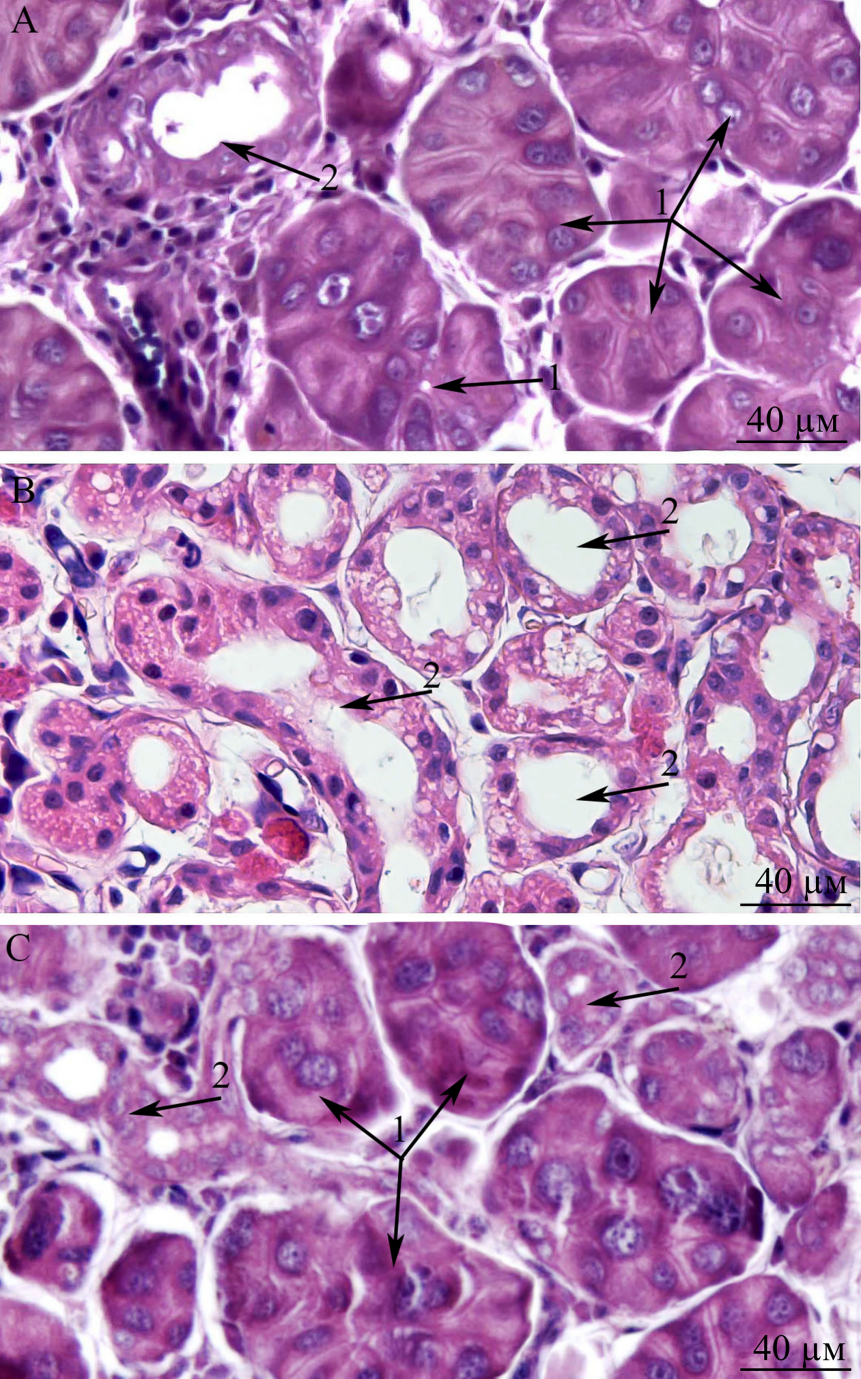
Histological structure of the lacrimal gland **A.** A fragment of a lobule from the lacrimal gland of a 3-month-old Wistar rat; **B.** a fragment of a lobule from the lacrimal gland of a 24-month-old Wistar rat; **C.** a fragment of a lobule from the lacrimal gland of a 24-month-old Wistar rat that was treated with the antioxidant SkQ1 (starting at age 1.5 months at the dose 250 nmol/ [kg body weight] daily). Arrows “1”: unaltered acini; arrows “2”: intercalary ducts. Staining with hematoxylin and eosin, magnification 400×.

This paper is a continuation of our preliminary study on 3-, 15-, and 24-month-old Wistar rats, where microscopic observation revealed ultrastructural deterioration of the lacrimal gland during aging [27]. Thus, it was interesting to examine age-related ultrastructural alterations of the lacrimal gland in more detail under the influence of the mitochondria-targeted antioxidant SkQ1. Here we shall first dwell on the possibility of quantitation of alterations in the lacrimal-gland ultrastructure directly on the sections during examination under an electron microscope. We must emphasize that these sections were comparable in size with histological sections. We analyzed not only ultrastructural changes in the lacrimal gland but also histological alterations in individual animals (Figure 10a-10c). The histological picture of the lacrimal gland that we observed in the same 24-month-old rats (Figure 10b) is in agreement with the literature data [2-6, 11, 12]. We then compared the results of ultrastructural analysis of lacrimal-gland tissue with the histological data in the same rats (Figure 10b). We saw that the regions of altered acini (according to histological analysis of aging-related changes) are actually regions filled with enlarged intercalary ducts with modified ultrastructure.

We observed various stages of ultrastructural deterioration of the ducts: from ducts with partially altered ultrastructure (Figure 5b) to those with completely altered epithelial cells (Figures 4a and 5a). According to our quantitation, the number of intact intercalary ducts in lacrimal-gland tissue of Wistar rats at this age decreased 12-fold in comparison with the 3-month-old rats. In most ducts of 24-month-old Wistar rats, the lining epithelial cells had electron-dense cytoplasm with difficult-to-distinguish cytoplasmic contents and numerous membrane-bounded granules (Figures 4a and 5a). In some cases, we could see that the granule contents were released into the lumen of the ducts (arrows “2” in Figure 4a). As a result, the duct lumen was filled with electron-dense granules, sometimes rather big ones. Apparently, such ultrastructure of the ducts represents the state known as duct blockage [5, 28]. In our opinion, it indicates the absence of functional activity of the lacrimal gland. Acini with native ultrastructure in old rats occurred rarely. According to our data, by age 24 months, in lacrimal-gland tissue of Wistar rats, the relative number of intact acini decreased 27-fold in comparison with 3-month-old rats. At the same time, remnants of degraded acinar cells were often seen among overgrown ducts (Figures 4a and 4b). Naturally, a significant reduction in tear secretion and deterioration of tear quality (similar to that in old people) were observed in 2-year-old rats [17].

Thus, our study shows that age-dependent changes in the lacrimal-gland structure of 24-month-old Wistar rats cannot be regarded as Harderian-gland-type metaplasia.

We also conducted morphometric analysis of electron-microscopic data from lacrimal-gland preparations (Figure 9). In SkQ1-treated rats, the number of sections of intercalary ducts showing signs of degradation decreased twofold. By contrast, the number of sections of acini showing signs of degradation decreased sevenfold, and the number of unaltered intercalary ducts was an order of magnitude greater in comparison with the untreated animals. The histological picture of the lacrimal gland of SkQ1-treated Wistar rats is in accord with the results of electron microscopy. In the histological samples of lacrimal-gland tissue from SkQ1-treated 24-month-old Wistar rats, the lacrimal gland appears as lobular structures, most of which were unaltered acini. Among them, we saw small intercalary ducts (Figure 10c).

It is worthwhile to consider the mitochondrial ultrastructure in lacrimal glands of Wistar rats. The finding that the ultrastructure of mitochondria in the 24-month-old rats had slight modifications is a surprise. Meanwhile, in acinar cells of the lacrimal gland of 24-month-old Wistar rats treated with SkQ1, we observed improvements in the mitochondrial ultrastructure. These improvements are similar to those observed in the retinal pigment epithelium of 11-month-old senescence-accelerated OXYS rats after 68-day treatment with eye drops of SkQ1 (Visomitin) [34] and in HeLa cells after incubation with 20 nM SkQ1 [35]. Instillation of Visomitin also increased tear production in OXYS rats (unpublished data). Our results may be explained as follows: the SkQ1-induced decrease in the level of endogenous ROS stimulates fusion of mitochondria and conjunction to the mitochondrial reticulum [35].

In both human and animal lacrimal glands, functional unit structure and secretory function begin to worsen in middle-aged individuals. With increasing age, a combination of events—leading to a continued decrease in secretion—causes DES with the aqueous deficiency (for review, see ref. [28]). It is thought that age 3 months in rats corresponds to the age of 16-18 years in humans; 12 months in rats to 40-50 years in humans; and 24 months to 65-85 years [3]. According to epidemiological data, age 50 years is precisely when the number of human patients with DES increases significantly, as does the number of treatment-resistant cases. The observed differences in the response to treatment of DES (with the same drug) among age groups of patients may be explained by different degrees of destructive changes in lacrimal-gland tissue. Evidently, the complete change in the ultrastructure of the majority of duct epithelial cells as well as the simultaneous degradation of acini become irreversible at some point. In such cases, artificial tear treatment is prescribed, which is typically used to alleviate DES symptoms. Our results indicate that dietary supplementation with the mitochondria-targeted antioxidant SkQ1 can significantly slow down the development of age-related deterioration of lacrimal-gland ultrastructure.

Overall, our data suggest that by age 24 months, in Wistar rats, ultrastructure of the lacrimal gland dramatically worsens, pointing to irreversible disturbances in this gland's functioning. Oral administration of SkQ1 starting at a young age significantly attenuated the age-related deterioration of the lacrimal gland. Our results are consistent with previous findings and indicate that effects of dietary supplementation with SkQ1 are clearly pleiotropic and are accompanied by inhibition of numerous typical traits of aging and by prevention of age-related diseases [29-32]. These include structural and functional aberrations that underlie DES.

## MATERIALS AND METHODS

### Animals, diet, and treatment

All experimental procedures were in compliance with the European Communities Council Directive of 24 November 1986 (86/609/EEC) and were approved by the Commission on Bioethics of the Institute of Cytology and Genetics (IC&G), Novosibirsk, Russia. Male Wistar rats were obtained from the Center for Genetic Resources of Laboratory Animals at the IC&G (RFMEFI61914X0005 and RFMEFI61914X0010). At the age of 4 weeks, the pups were weaned, housed in groups of five animals per cage (57 × 36 × 20 cm), and kept under standard laboratory conditions (22 ± 2°C, 60% relative humidity, and 12-h light/12-h dark cycle. The animals were provided with standard rodent feed (PK-120-1; Laboratorsnab, Ltd., Moscow, Russia) and water *ad libitum*. To assess the influence of oral SkQ1 (from age 1.5 to 24 months) on the ultrastructure of the lacrimal gland, we randomly assigned 1.5-month-old rats to one of two groups (five rats per group). One group consumed a control diet, while the other consumed the same diet supplemented with 250 nmol SkQ1 per kilogram of body weight per day. SkQ1 was synthesized as described earlier [22].

At age 24 months, the SkQ1-treated and untreated rats were euthanized by CO_2_ asphyxiation; the lacrimal glands were carefully excised for subsequent analyses, with 3-month-old rats serving as an additional control (five rats per group).

### Morphological analysis of the lacrimal gland

For this purpose, pieces of lacrimal glands were fixed with Bouin's fluid, briefly washed in distilled water, and dehydrated in 70%, 95%, and 100% ethanol. After that, the tissue pieces were embedded in paraffin and used to prepare serial sections 4-5 μm thick. The slices were stained with hematoxylin and eosin.

### Electron microscopy

Samples of lacrimal glands were fixed with a 3% glutaraldehyde solution (pH 7.4) for 2 h at 4°C, then overfixed with a 1% osmium tetroxide solution for 1.5 h and dehydrated in solutions containing increasing alcohol concentrations (70% alcohol was saturated with uranyl acetate). The samples were then embedded in Epon-812 epoxy resin. Serial ultrathin sections were made on a Leica ULTRACUT UCT microtome and stained with lead according to the Reynolds method [33]. The resulting slices were scanned and photographed using an JEM-1400 electron microscope (Jeol, Japan).

### Calculations

For morphometric analysis of the lacrimal-gland tissue, the volume share of the acini and volume share of intralobular ducts were assessed by direct counting during examination under the electron microscope. The numbers of acini and ducts were calculated within the area of 230633 μm^2^ or 0.23 mm^2^. In each group of the rats, ~400 of such areas were processed this way.

For morphometric analysis of mitochondrial ultrastructure and of the average area of one mitochondrial section in the lacrimal gland, the Adobe^®^ Photoshop^®^ graphics editor was used for direct calculations, as described elsewhere [36-37].

Before statistical analysis, the resulting quantitative data were analyzed (for the type of distribution of random values) by the Kolmogorov-Smirnov test. Further data analysis was conducted by nonparametric methods, and statistical significance of the differences was evaluated by the Mann-Whitney *U* test. The Statistica 10 software was used for all calculations.
